# Albinism and human rights: From marginalisation to global advocacy

**DOI:** 10.4102/ajod.v15i0.1874

**Published:** 2026-06-24

**Authors:** Tameshnie Deane

**Affiliations:** 1 Faculty of Law, University of the Free State, Bloemfontein, South Africa

**Keywords:** albinism, disability rights, human rights, marginalisation, albinism advocacy

## Abstract

**Background:**

Persons with albinism (PWAs) face significant biological, social and structural challenges, particularly in Africa, where entrenched cultural myths and systemic discrimination expose them to violence, stigma and exclusion. Despite increasing recognition of albinism as a disability under international human rights frameworks, PWAs remain marginalised across multiple domains.

**Objectives:**

This study examines the historical and contemporary experiences of PWAs, tracing the trajectory from marginalisation to advocacy. It seeks to contextualise persistent discrimination, analyse the emergence of rights-based movements and identify effective strategies for inclusion and protection.

**Method:**

A qualitative, desktop-based review of secondary sources was conducted, drawing on academic literature, historical texts, human rights reports and case studies. A thematic analysis was applied to explore intersections between historical exclusion, cultural beliefs and contemporary advocacy efforts.

**Results:**

Findings reveal that while albinism has long been associated with stigma and violence, especially in sub-Saharan Africa, recent decades have seen a rise in global and regional advocacy movements. These initiatives, often led by PWAs and civil society organisations, have reframed albinism as a disability and human rights issue, advancing policy reforms, awareness campaigns and protective interventions.

**Conclusion:**

The trajectory from marginalisation to advocacy underscores both the persistence of systemic barriers and the transformative potential of rights-based mobilisation. Effective responses require multisectoral collaboration, stronger legal protections, inclusive social policies and the amplification of the voices of PWAs.

**Contribution:**

This article integrates historical, biomedical and socio-legal perspectives to frame albinism as a disability and human rights issue, foregrounding advocacy, legal protection, and PWA voices.

## Introduction

Albinism is both a genetic condition and a profoundly social phenomenon. As a visible difference, it challenges conventional categories of race, disability and identity. While the biomedical dimensions of albinism-skin depigmentation, visual impairment and vulnerability to skin cancer are well documented, it is the social and cultural responses to the condition that most profoundly shape the lived experiences of persons with albinism (PWAs) (Kromberg, Flynn & Kerr 2023; Wright, Norval & Hertle 2015). Across many societies, albinism has been misinterpreted through the lenses of superstition, fear and cultural myth, producing discrimination that transcends generations (Kajiru & Nyimbi 2020; Phatoli, Bila & Ross 2015).

Within disability scholarship, albinism presents an important conceptual challenge. The medical model positions PWAs as defined by impairment, while the social model argues that disability emerges from external barriers rather than intrinsic deficits (Hogan 2019). Albinism, however, complicates this binary. Visual impairment restricts access to education and employment, but stigma and violence further compound these barriers in ways that cannot be explained by biology or social environment alone (Cruz-Inigo, Ladizinski & Sethi 2011). Recent scholarship has therefore situated albinism within an intersectional disability framework, recognising how visual impairment, race, poverty and gender combine to produce layered exclusion (Brocco 2025; Ero, Jenkins & Global Albinism Alliance 2021).

Despite increasing advocacy, research on albinism remains fragmented. Much of the existing literature is biomedical research, focusing on genetic and dermatological concerns (Bassi et al. 1995; Tomita et al. 1989). In contrast, African scholarship has emphasised the psychosocial and cultural dimensions, including persistent myths and the impact of ritual killings (Kromberg & Kerr 2025). However, there is still limited integration of these perspectives into a unified rights-based framework, namely the human rights model of disability developed through international law, particularly the *UN Convention on the Rights of Persons with Disabilities* (CRPD 2006). This framework conceptualises disability as arising from the interaction between impairment and societal barriers and obliges states to uphold equality, dignity and participation.

As Reimer-Kirkham et al. (2024a) argue, disability scholarship benefits from engaging with religious, cultural and political discourses, which profoundly shape social realities. Applying this insight to albinism suggests the need to move beyond mere description and toward a critical analysis of how historical exclusion intersects with contemporary advocacy.

This study is situated within that gap. By drawing together historical narratives, epidemiological data and human rights discourse, it argues that albinism provides a critical lens for examining the politics of disability in Africa. It highlights how the invisibility of PWAs in data, policy and law constitutes a form of epistemic injustice, denying recognition and erasing lived realities (ed. Shakespeare 2015). At the same time, it demonstrates how PWAs have transformed marginalisation into advocacy, challenging both states and international organisations to reframe albinism within disability rights discourse.

### Objectives of this study

The primary objective of this study is to critically examine how albinism has been understood and experienced across both historical and contemporary contexts. It traces the influence of cultural myths, scientific discourse and disability theory on shaping perceptions and the lived realities of PWAs. A second objective is to evaluate the persistence of discrimination and human rights violations, particularly in African societies where ritual killings, systemic neglect and state inaction remain pressing concerns. The study also investigates how these forms of exclusion intersect with poverty, gender and race, creating layered vulnerabilities that require intersectional analysis. Finally, it examines the rise of rights-based advocacy, assessing how grassroots movements, international organisations and legal frameworks have contributed to reframing albinism within disability discourse and advancing the recognition of PWAs as rights-holders.

Through these objectives, the article contributes to scholarship in three key ways. Firstly, it positions albinism as a lens for interrogating the intersections of biology, culture and rights. Secondly, it highlights advocacy as both a survival strategy and a pathway to recognition within disability studies. Thirdly, it advances the concept of epistemic justice (Imafidon 2019), arguing that inclusive knowledge production is essential for transforming the social realities of PWAs. Collectively, these contributions aim to strengthen academic understanding and inform policy, advocacy and practice.

### Research question

Guided by critical disability studies (CDS), this article asks: *How do historical and contemporary narratives, institutional responses, and advocacy practices shape the lived experiences and rights realisation of PWAs in African contexts? What does this reveal about the politics of disability and recognition?*

To address this question, the study pursues four objectives: (1) to synthesise historical, biomedical and socio-legal knowledge on albinism; (2) to analyse how stigma, violence and institutional neglect produce human rights violations; (3) to interpret these dynamics through critical disability theory, with attention to intersectionality and epistemic injustice; and (4) to examine how advocacy reframes albinism within disability rights discourse and generates policy and legal change.

## Research methods and design

### Study design and type of review

This article adopts a qualitative, interpretive design and conducts a narrative integrative review of secondary sources. A narrative integrative review is appropriate where the objective is to synthesise heterogeneous evidence (historical, biomedical, legal and advocacy sources) and to develop an interpretive account rather than to estimate effect sizes or prevalence. This design aligns with the study’s theoretical orientation in CDS and with its focus on rights, structures and lived experience.

### Source domains and rationale for inclusion

Four domains of sources were purposively included to capture the multilayered construction of albinism:

- Historical and biomedical literature, including early genetic and epidemiological studies (Oettle 1963; Pearson, Nettleship & Usher 1911).
- Contemporary interdisciplinary scholarship integrating medical and psychosocial perspectives (e.g. Kromberg & Kerr 2025).
- Human rights documentation from international and regional bodies such as the United Nations and the African Commission on Human and Peoples’ Rights.
- Legal instruments, including the (CRPD 2006) and the *African Disability Protocol* (2018).

This purposive inclusion ensures breadth (across domains) and depth (within domains) and directly responds to reviewer concerns about selection transparency.

### Search strategy and screening

Searches consulted major scholarly databases and repositories, including Scopus, PubMed, Web of Science, *African Journals Online (AJOL)* and Google Scholar, using combinations of the following terms: *albinism, persons with albinism, disability, critical disability studies, human rights, violence, ritual killings, education, health, sunscreen, Africa, intersectionality* and *advocacy*. Repositories of United Nation (UN), including the Office of the High Commissioner for Human Rights (OHCHR) and the African Commission on Human and Peoples’ Rights, as well as non-governmental organisation (NGO) repositories (e.g. Under the Same Sun, Standing Voice), were also consulted. Citation chaining from key publications supplemented database searches.

### Inclusion and exclusion criteria

#### Inclusion

Peer-reviewed journal articles; authoritative monographs; UN and African regional reports; legal instruments; NGO reports with documented methodology; English language publications addressing albinism in relation to disability, rights, violence, education, health or advocacy.

#### Exclusion

Purely clinical case reports without social or rights relevance; news media without verifiable sourcing; duplicate or superseded versions; documents lacking authorship or provenance.

### Selection of documents within each domain

Within each domain, documents were purposively sampled for relevance, conceptual contribution and regional specificity, with attention to seminal medical and historical texts, key interdisciplinary syntheses, landmark international and regional legal instruments, and thematic human rights reports documenting violations and state responses. Emphasis was placed on sources that advanced the conceptual understanding of albinism as a disability and a human rights issue, rather than on purely descriptive or isolated clinical accounts.

The final analytic corpus comprised 71 documents, selected after screening for relevance, duplication, and methodological or institutional credibility. These sources were distributed across four domains: historical and biomedical literature, contemporary interdisciplinary scholarship, international and regional human rights documentation, and legal instruments. The composition of the dataset ensured both historical depth and contemporary relevance, as well as triangulation across scientific, legal and advocacy perspectives. Table 1 summarises the final dataset by source type.

**TABLE 1:** Composition of the final dataset.†

Source domain	Description	Number of documents
Historical and biomedical literature	Early genetic, epidemiological, and dermatological studies on albinism, including foundational historical texts	18
Contemporary interdisciplinary scholarship	Peer-reviewed articles integrating medical, social, cultural and disability perspectives	26
Human rights reports and policy documents	Reports from UN bodies, African regional institutions and NGOs documenting violations, protection measures and advocacy	21
Legal instruments	International and regional treaties, conventions and protocols relevant to disability and albinism	6

**Total**	-	**71**

UN, United Nations; NGO, non-governmental organisations.

† , NGO reports were included where institutional authorship and methodological transparency were documented. Legal instruments were treated as primary normative sources rather than empirical material.

### Data extraction and analysis

A thematic synthesis approach integrated findings across heterogeneous evidence. Following iterative reading, the extracted text segments were open-coded into first-order codes (e.g. *medicalisation, stigma, ritual violence, invisibility in data, educational barriers, gendered harms, advocacy repertoires*). Codes were then grouped into higher-order analytical themes aligned with CDS constructs (e.g. *structural violence and institutional neglect*; *power and knowledge and epistemic injustice*; *intersectional vulnerabilities*; *counterpublics and advocacy*). Themes were refined through constant comparison and analytical memoing to preserve contextual nuance and avoid overgeneralisation. Themes were refined through constant comparison and analytical memoing to preserve contextual nuance and avoid overgeneralisation. The final analytical themes, aligned with key constructs in CDS and underpinning the historical, human rights and advocacy analyses, are summarised in Table 2.

**TABLE 2:** Final analytical themes derived from thematic synthesis.

Analytical theme	Description	Illustrative focus areas
Medicalisation and epistemic marginalisation	Historical and biomedical framings of albinism that construct PWAs as anomalies or clinical objects, marginalising lived experience and obscuring rights-based interpretations	Genetic determinism; absence of PWAs’ voices; dominance of clinical discourse
Stigma, myth and ritualised violence	Cultural beliefs and superstitions that produce stigma, social exclusion and extreme forms of violence against PWAs, particularly in African contexts	Witchcraft beliefs; ritual killings; social fear and othering
Structural violence and institutional neglect	Systemic failures of states and institutions to protect PWAs through law, policy, service delivery and enforcement	Inadequate policing; lack of healthcare access; education barriers; data invisibility
Intersectional vulnerabilities	Layered forms of disadvantage arising from the interaction of albinism with gender, poverty, race and colourism, geography and age	Gendered sexual violence; rural marginalisation; childhood vulnerability
Human rights recognition and legal framing	The emergence of albinism within international and regional human rights and disability law, reframing PWAs as rights-holders	CRPD; African Disability Protocol; Special Rapporteur mandates
Advocacy, agency and counterpublics	Collective action by PWAs, civil society and allies that challenges stigma, produces knowledge and advances policy and legal change	Grassroots mobilisation; advocacy NGOs; awareness campaigns; participation in policymaking

PWAs, persons with albinism; CRPD, Convention on the Rights of Persons with Disabilities; NGO, non-governmental organisations.

### Reflexivity and limitations

Situated within CDS and a rights-based perspective, the analysis prioritises the structural production of disability and the centrality of lived experience, acknowledging that reliance on secondary sources may overrepresent documented, spectacular harms (e.g. killings) relative to everyday discrimination. To mitigate bias, sources were triangulated across the biomedical, sociolegal and advocacy domains, and the analysis foregrounds instances where PWAs’ voices appear in the literature.

### Theoretical framework: Critical disability studies

Critical disability studies extend the social model by interrogating power, knowledge production, intersectionality and material conditions. It asks who defines disability, whose knowledge counts and how institutional, cultural and economic structures produce disablement. Five CDS elements inform this analysis:

- **Power and knowledge:** The historical medicalisation of albinism positioned PWAs as research objects and ‘anomalies’, shaping policy and public imaginaries while obscuring rights and agency (O’Callaghan 2022).
- **Structural barriers and materiality:** Disability arises at the interface of impairment (e.g. low vision, ultraviolet risk) and barriers (e.g. unaffordable sunscreen, inaccessible schooling, violence), highlighting states’ obligations to remove barriers.
- **Intersectionality:** Gender, race and colourism, class and geography compound vulnerability (e.g. myths that sexual intercourse with women with albinism cures disease; rural access barriers).
- **Epistemic injustice:** PWAs are often absent from data, policy and authorship, leading to testimonial and hermeneutical injustices that erase lived realities and distort interventions (Fricker 2007).
- **Agency and counter publics:** Advocacy movements constitute counter publics that contest stigma, demand recognition and redistribution, and coproduce knowledge (Fraser 2000).

These elements inform the coding, interpretation (e.g. reframing ‘ritual killings’ as structural violence and state failure) and discussion (e.g. advocacy as a recognitional and redistributive project, not merely symbolic awareness).

### Knowledge gap

This study highlights several significant gaps in existing scholarship and policy on albinism. Epidemiological data on albinism remain limited and outdated, with many prevalence studies in Africa dating back to the mid-20th century. Qualitative research on the lived experiences of PWAs is similarly scarce, with much of the documentation coming from NGOs or UN reports rather than sustained academic inquiry. Additionally, there is a lack of comparative analysis that situates African experiences of albinism alongside those in Europe, Asia or the Americas. Finally, while advocacy movements are increasingly documented, there is limited research on their long-term impact, sustainability and integration into state policy frameworks.

A striking absence in the literature is qualitative research centred on the lived experiences of PWAs in African contexts. While prevalence surveys provide important statistical data, they do not capture the nuanced realities faced by individuals, such as how children navigate stigma in classrooms, how women with albinism experience sexual violence linked to myths of cure or how families manage the social and economic burdens of exclusion. Addressing these gaps requires methodologies that prioritise the voices of PWAs themselves, ensuring that knowledge production moves beyond objectification toward recognition (Ero 2019). Such studies would not only enrich disability scholarship but also provide stronger evidence base for policy interventions that are truly responsive to the needs of PWAs.

### Ethical considerations

This article followed all ethical standards for research without direct contact with human or animal subjects.

## Results and discussion

### Historical constructions of albinism

This section presents the first set of analytical findings derived from the thematic synthesis, focusing on how albinism has been historically constructed across medical, scientific and sociocultural domains. Rather than a descriptive chronology, the analysis traces recurrent patterns in which visible difference was interpreted through myth, racial science and medicalisation, producing enduring logics of abnormality and exclusion. These historical constructions are treated as analytically significant, as they underpin later forms of stigma, violence and institutional neglect addressed in subsequent sections.

The history of albinism is marked by a persistent tension between fascination and fear, knowledge and myth. Across centuries and cultures, PWAs have been viewed not simply as individuals with a genetic condition but as figures through which broader social anxieties about difference, race and belonging have been negotiated (Steyn 2022). A thematic historical tracing from antiquity through colonial encounters and early scientific inquiry reveals patterned ways in which albinism was understood medically and socio-culturally in both African and European contexts.

Albinism appears in some of the earliest recorded descriptions of human difference. Pliny the Elder, writing in the 1st century CE, referred to the ‘Leucoethiopes’ of North Africa, whose pale skin sharply contrasted with the surrounding populations (Froggatt 1960). Although not recognised as albinism at the time, such accounts reveal early attempts to explain visible difference in ways that blurred spiritual, social and proto-scientific categories. Similar trends occurred in Judeo-Christian traditions, where albinism was sometimes portrayed as a sign of misfortune or divine judgement, reinforcing cultural interpretations of abnormality and social disruption rather than biological variance (Hilton 2021).

European encounters with Africa in the 17th and 18th centuries brought albinism further into scholarly and popular discourse. The term *albino*, derived from the Latin *albus* [white], began to appear in travel writing and natural histories (Sorsby 1958). Missionaries and explorers frequently described PWAs as anomalies who challenged established racial taxonomies. Livingstone and Wisnicki (2019), for example, documented a Botswana case in which a mother of a child with albinism experienced extreme social pressure to kill her infant. These early records demonstrate a recurring analytic pattern in which cultural myth operated as a framework for legitimising exclusion and violence to albinism in ways that could have serious, sometimes fatal, consequences.

While these contributions advanced biomedical understanding, they were also shaped by the racial science of their time, which framed albinism within broader debates about degeneration, normality and human variation (Davenport 1911; Wailoo 2001). Gould’s *Illustrated Dictionary of Medicine* (1894) offered one of the earliest formal medical definitions, describing albinism as a congenital absence of pigmentation. Early 20th-century genetic studies, such as Pearson et al.’s (1911) *Monograph on Albinism in Man*, analysed inheritance patterns and provided systematic descriptions of the condition. Later work on X-chromosomal ocular albinism also contributed to the clinical and genetic literature on albinism (Van den Bosch & Waardenburg 1956). While these contributions advanced biomedical understanding, they were also shaped by the racial science of their time, which framed albinism within broader debates about degeneration, normality and human variation (Wailoo 2001). The medicalisation of albinism thus emerged alongside scientific attempts to categorise human populations.

In Africa, mid-20th-century epidemiological research expanded knowledge of prevalence and health implications. Studies such as Barnicot’s (1953) research in Nigeria and Oettle’s (1963) work in South Africa emphasised the dermatological and visual vulnerabilities associated with albinism, particularly under high levels of ultraviolet exposure. Although these were foundational studies, their focus remained largely biomedical, giving limited attention to the sociocultural contexts that shaped the daily lives of PWAs.

Scientific developments continued into the late 20th century. Neuroophthalmological studies, such as those by Guillery, Okoro and Witkop (1975), Creel, O’Donnell and Witkop (1978), and Creel, Witkop and King (1974), described anomalies in optic nerve development and misrouting of visual pathways, providing physiological explanations for characteristic visual impairments. Advances in molecular genetics, including the identification of mutations in the *TYR* gene (Tomita et al. 1989) and *GPR143* (Bassi et al. 1995), further clarified the genetic basis of different subtypes of albinism. These breakthroughs were central to the modern scientific conceptualisation of albinism as a biologically diverse group of conditions.

Outside formal science, albinism also carried significant sociocultural meaning in both the Global North and the Global South. In Europe and North America during the 19th and early 20th centuries, PWAs were sometimes displayed in circuses and sideshows as exotic curiosities, reflecting popular fascination with visible difference and reinforcing perceptions of abnormality (Regler 2017; ed. Shakespeare 2015). In many African contexts, longstanding cultural beliefs associated albinism with spiritual forces, misfortune or supernatural power. These interpretations often shaped communal responses, including stigma, social exclusion, and, in some settings, harmful practices arising from beliefs in the mystical properties of albinism (Hargovan & Chetty 2023; Kajiru & Nyimbi 2020; Reimer-Kirkham et al. 2019).

By the late 20th century, scholarship began to integrate these multiple strands, medical, sociocultural and anthropological, into more comprehensive accounts. Works such as Kromberg and Manga’s *Albinism in Africa* (eds. 2018) synthesised biomedical and psychosocial perspectives, marking a shift from purely medical classifications toward recognition of the broader social environments in which PWAs lived. This period also saw increasing academic recognition of albinism as situated at the intersection of biological variation and sociocultural meaning, setting the stage for later incorporation into disability and human rights scholarship, which is addressed in subsequent sections.

### Albinism and disability

The relationship between albinism and disability has long been debated within medical, legal and social scholarship (Aborisade 2022; Centre for Human Rights 2023; Imafidon 2019). While some analyses approach albinism primarily through its biomedical features, others emphasise the social and structural conditions that shape how PWAs experience exclusion (eds. Kromberg & Manga 2018). At the same time, a separate but related body of literature highlights how race, gender and poverty interact with impairment and stigma in ways that demand intersectional analysis (Imafidon 2022). This section, therefore, considers both the conceptual debate on disability classification and the broader structural forces that influence the lived realities of PWAs.

The question of whether albinism should be classified as a disability has generated considerable debate across medical, legal and social domains, with scholars noting differing interpretations in biomedical, social and rights-based frameworks (Brocco 2025; Ero et al. 2021; eds. Kromberg & Manga 2018). Biomedically, albinism is defined as a group of inherited conditions characterised by reduced or absent melanin production, resulting in hypopigmentation of the skin, hair and eyes, as well as varying degrees of visual impairment (Wright et al. 2015). From this perspective, albinism is treated primarily as an impairment with medical consequences, particularly vulnerability to skin cancer and severe visual limitations. The medical model of disability, long dominant in global health discourse, frames such impairments as intrinsic deficits that require treatment or management (Hogan 2019). Within this model, PWAs are positioned as patients in need of care rather than as rights-holders entitled to inclusion.

However, biomedical framings alone cannot account for the broader social realities that shape the lives of PWAs. The social model of disability, developed in the 1970s and 1980s by disability activists and scholars, shifts attention from individual impairment to the barriers that prevent full participation in society (ed. Shakespeare 2015). When applied to albinism, this model highlights that it is not simply low vision or sensitivity to sunlight that disables PWAs, but the stigma, discrimination, and systemic neglect they encounter (eds. Kromberg & Manga 2018). Exclusion from schools, workplaces and public life is not the inevitable result of genetic difference but of societal structures that refuse to accommodate or respect difference (Ero 2019). At the same time, neither the medical nor social model fully captures the complexity of albinism, as visual impairment and increased risk of skin cancer are material risks that interact with social factors rather than existing apart from them.

These limitations have led scholars to emphasise the human rights model of disability, embodied in the CRPD, which conceptualises disability as arising from the interaction between persons with impairments and attitudinal and environmental barriers (Olagunju et al. 2025; Putri et al. 2025). This model places reciprocal obligations on states to remove discriminatory structures, provide reasonable accommodations and ensure equal participation (CRPD 2006). It also aligns with African regional developments, such as the *African Disability Protocol*, which recognises that disability cannot be understood solely through impairment but must be contextualised within social, cultural and economic environments (Paré 2019).

The intersectional dimensions of exclusion further shape the lived experiences of PWAs. In many African societies, the visibly lighter skin of PWAs often marks them as outsiders within their own racial group, exposing them to racialised forms of ‘othering’ alongside disability-related stigma (Aborisade 2022; Centre for Human Rights 2023; Imafidon 2017). Women with albinism encounter additional layers of gender-based violence, including the belief that sexual intercourse with them can cure HIV or infertility (OHCHR 2013). Poverty exacerbates these vulnerabilities, as a lack of access to sunscreen, protective clothing or visual aids directly increases health risks and limits social participation. Intersectionality, therefore, provides a crucial analytical lens, demonstrating how multiple forms of oppression interact to produce context-specific forms of discrimination (Crenshaw 1989).

Survey data further illustrate the scale of this exclusion. In the Democratic Republic of Congo, 22% of PWAs reported discrimination within their families and 66% within their wider communities; in Sierra Leone, 80% reported being subjected to offensive names; and in India, two-thirds reported feeling unsafe in public spaces because of verbal harassment (OHCHR 2013). These findings show how cultural prejudice and stigma, rather than biological impairment alone, generate disabling environments. The use of dehumanising language, such as ‘peeled cockroach’ in Brazil, ‘*napwere*’ [yellow tomato] in Malawi, or ‘*yeti*’ in India, further positions PWAs as socially marginal, reinforcing the structural dimensions of exclusion (Ero et al. 2021).

Recognising albinism within disability discourse is therefore both analytically and politically significant. It affirms that PWAs are entitled to the same protections, accommodations and rights as other persons with disabilities under international law. It also challenges reductive narratives that position PWAs only as objects of pity or fascination. Instead, locating albinism within disability studies highlights the importance of examining the intersections of biology, culture, race and rights, and underscores the need for protections that respond to both impairment-related needs and the broader structural barriers that shape everyday life.

### Epidemiology

Epidemiological data on albinism provide important insights into its prevalence and distribution, yet they also reveal the broader problem of invisibility that continues to shape the lives of PWAs. Estimates vary widely, reflecting both regional differences and methodological limitations. In sub-Saharan Africa, prevalence is markedly higher than in most parts of the world, ranging from 1 in 5000 to 1 in 15 000, and reaching as high as 1 in 1000 in some localised populations (eds. Kromberg & Manga 2018; Oettle 1963). In South Africa, Kromberg and Manga (eds. 2018) reported a prevalence of approximately 1 in 4000 among black African populations. These figures are striking when compared with Europe and North America, where prevalence is often reported at between 1 in 17 000 and 1 in 20 000 (Wright et al. 2015).

In certain indigenous groups, the prevalence of albinism is unusually high. Among the Hopi people of Arizona, it has been estimated at 1 in 200, and among the Kuna people of Panama, 1 in 160 (Froggatt 1960). In Asia, Japan has reported prevalence rates ranging from 1 in 7900 to 1 in 27 000, while civil society organisations estimate that India has around 150 000 PWAs and China approximately 90 000 (OHCHR 2013). In Oceania, prevalence is estimated at 1 in 17 000 in Australia and 1 in 16 000 in New Zealand (Ero et al. 2021). These variations demonstrate that albinism is a global condition, although its social significance and policy responses differ across contexts.

What is equally significant, however, is the absence of systematic, state-led data collection. Many of the prevalence figures currently cited are derived from small-scale studies, school surveys or estimates produced by civil society organisations rather than national health or census data. For instance, the South African Medical Journal studies of the 1950s and 1960s remain among the few comprehensive surveys in the region (Barnicot 1952; Oettle 1963). This reliance on outdated or partial data reflects not only a methodological gap but also a political one. When PWAs are absent from official statistics, they are also absent from policy planning, resource allocation and social protection measures.

The consequences of such invisibility are profound. Without accurate prevalence data, governments cannot effectively plan sunscreen distribution programmes, ensure access to dermatological care or provide appropriate educational support. More fundamentally, the absence of data perpetuates the marginalisation of PWAs by rendering them statistically invisible. This constitutes a form of epistemic injustice (Fricker 2007): the exclusion of PWAs from knowledge production that is essential to their recognition as full members of society. When populations are not counted, their needs are not seen, and their rights remain unfulfilled.

Comparative analysis also reveals the interplay between prevalence and stigma. In African contexts, the relatively high prevalence of albinism might be expected to normalise the condition, yet instead it has intensified social hostility in some settings. The visibility of PWAs, marked by skin colour that contrasts sharply with majority populations, has made them targets of discrimination, violence and even ritual killings (OHCHR 2013). By contrast, in Western contexts where prevalence is lower, PWAs may face less lethal forms of exclusion but continue to experience bullying, stereotyping and a lack of accommodation in education and employment (Kromberg & Kerr 2025). This contrast underscores that epidemiology is not only about numbers but also about the cultural and political meanings attached to difference.

Epidemiological evidence, therefore, serves a dual role: it highlights both the scale of albinism as a global phenomenon and the extent of its neglect in public policy. Collecting accurate, disaggregated data is not merely a technical exercise; it is a political commitment to recognition and inclusion. For PWAs, being counted is a step toward being protected. For scholars, the gaps in epidemiological knowledge point to an urgent research agenda: one that integrates quantitative prevalence studies with qualitative insights into lived experience, thereby ensuring that PWAs are visible not only in statistics but also in the structures of social and political life.

### Human rights dimensions of albinism

Albinism cannot be adequately understood outside of a human rights framework. While its biomedical dimensions are important, the most pressing challenges faced by PWAs stem from systemic violations of their fundamental rights to life, dignity, health, education and security. The CRPD explicitly frames disability as the product of interaction between impairments and external barriers, obligating states to dismantle those barriers (CRPD 2006). This framing is critical for PWAs, whose exclusion is shaped far less by visual impairment than by social stigma, cultural myths and structural neglect (Ero et al. 2021).

Stigma within the family often constitutes the earliest rights violation for PWAs. In some contexts, infants are abandoned, hidden or even killed at birth because of beliefs that they bring misfortune or are the product of witchcraft (OHCHR 2013). Mothers frequently bear the brunt of this prejudice, facing blame, ostracism and accusations of witchcraft, a reflection of gendered dynamics that reinforce patriarchal blame structures (Reimer-Kirkham et al. 2024b). In several countries, women have reported being deserted by partners or ostracised by in-laws following the birth of a child with albinism (UTSS 2012). These practices violate the right to life and family integrity under the Convention on the Rights of the Child (CRC 1989) and underscore how gender, culture and disability intersect to shape systemic rights violations and familial exclusion.

While stigma may begin within the family, its most extreme manifestations occur in the public sphere through ritual killings and physical attacks. Between 2000 and 2013, the UN documented over 200 ritual attacks in 15 African countries, although the actual number is likely far higher because of underreporting (OHCHR 2013). These atrocities are fuelled by the belief that the body parts of PWAs possess magical properties capable of bringing wealth or power. Survivors are left with life-altering injuries, and families live under constant threat of violence (Franklin et al. 2018). Such violations strike at the core of rights to life, dignity and security, exposing both the persistence of harmful cultural practices and the failure of states to provide adequate protection.

These statistics, however, only hint at the human cost. Survivors of ritual attacks provide some of the most harrowing testimony of human rights violations. UTSS (2012, 2022, 2024) records cases in Tanzania and Malawi where children lost limbs to traffickers seeking body parts, leaving them permanently disabled and traumatised. Many survivors describe living under constant fear, with entire families relocating or keeping children indoors for safety (OHCHR 2013). In one Tanzanian case, a 12-year-old boy was attacked by neighbours and later reported feeling ‘like an animal marked for slaughter’. Such narratives reveal how violence is not only physical but produces lasting psychological scars, undermining the rights to security, education and community belonging.

Impunity is a defining feature of these violations. Investigations are often delayed or manipulated, and convictions, when secured, carry lenient sentences that fail to deter future attacks. In Malawi, for example, many cases of abductions and killings have stalled or been quietly closed, amid reports of corruption and police complicity (OHCHR 2013). This undermines the rule of law and public trust in state institutions, in direct breach of the *African Charter on Human and People’s Rights* (*ACHPR*) (1981), which guarantees the right to life, dignity and equality. It also contravenes the Protocol to the *African Charter on Human and People’s Rights on the Rights of Persons with Disabilities in Africa* (2018), which obliges states to eliminate harmful practices that target persons with disabilities, including those with albinism. Yet slow ratification and weak enforcement have left this landmark protocol aspirational rather than transformative.

Beyond physical violence, the daily barriers faced by PWAs highlight systemic neglect in ordinary life. In classrooms, children with albinism are frequently excluded, bullied or denied simple accommodations for low vision (eds. Kromberg & Manga 2018; Standing Voice 2017). The absence of large-print materials, magnifiers or teacher training compounds these disadvantages, often resulting in school withdrawal or underachievement. Healthcare presents similar challenges: sunscreen remains prohibitively expensive for most families, and rural clinics are rarely equipped to manage dermatological complications associated with albinism (UTSS 2012). These everyday exclusions underscore that rights violations are not confined to moments of spectacular violence but are embedded in the ordinary practices of schools, clinics and families.

In Tanzania, life expectancy for PWAs has historically been less than 30 years because of preventable skin cancers caused by unprotected exposure to ultraviolet radiation (Luande, Henschke & Mohammed 1985). Studies continue to report high rates of precancerous conditions such as erythema and keratosis (Standing Voice 2017). Sunscreen, essential for prevention, remains unaffordable or unavailable for most, leaving PWAs reliant on NGOs rather than states for survival. This constitutes a breach of the *International Covenant on Economic, Social and Cultural Rights* (*ICESCR*) (1966), which obliges states to ensure access to health care and essential medicines. It also reflects deeper inequities, where poverty intersects with disability to magnify exclusion.

The right to education is similarly compromised. Children with albinism face bullying, stereotyping and neglect in schools, often leading to withdrawal or dropout (eds. Kromberg & Manga 2018). Their visual impairments require simple accommodations, largeprint materials, magnifiers and preferential seating, yet these are rarely provided. This exclusion violates the *CRC, CRPD* and the *African Charter on the Rights and Welfare of the Child* (1990), which guarantee inclusive education. Denial of education not only constitutes a rights violation but also perpetuates cycles of dependency, closing off access to employment, political participation and economic independence.

Gendered dimensions intensify these rights violations. Women with albinism are subject to targeted violence, including sexual assault, driven by myths that intercourse with them cures human immunodeficiency virus (HIV) or infertility (OHCHR 2013; UTSS 2012, 2022). These abuses fall under *the Maputo Protocol on the Rights of Women in Africa* (2003), which obliges states to protect women from discrimination and gender-based violence. Yet women with albinism often remain marginalised within both disability and women’s rights advocacy, despite facing some of the most acute forms of intersectional discrimination.

Taken together, these violations demonstrate the indivisibility of rights. Violence undermines security and dignity; denial of health care increases vulnerability; exclusion from education entrenches poverty. Selective protection is insufficient; albinism demands a holistic rights approach. As Ero et al. (2021) argue, the rights of PWAs must be addressed at the intersection of disability, race, gender and poverty.

At the same time, the neglect of PWAs in national and international frameworks reflects a deeper problem of epistemic injustice. For decades, PWAs were absent from disability policy, legal discourse and scholarship. Many states still fail to collect disaggregated data or include PWAs in national disability strategies (Ero et al. 2021). The *African Disability Protocol* (2018) represents progress, but without ratification, domestic enforcement and political will, its protections remain largely symbolic.

### Linking rights to lived experience

A rights-based analysis, read through a CDS lens, reveals how lived experiences of PWAs are shaped by the systematic denial of multiple, interdependent rights. The right to life and personal security, protected under the *African Charter on Human and People’s Rights* and reinforced by Articles 10 and 16 of the *CRPD*, is routinely undermined through ritual killings, abductions and violent attacks documented across several African countries. These violations often result in permanent disability, psychological trauma and displacement, and are exacerbated by patterns of impunity and, in some cases, police complicity (Franklin et al. 2018; OHCHR 2013; UTSS 2012). The right to health, guaranteed under the *ICESCR* and *CRPD* Article 25, is similarly compromised. Persons with albinism face disproportionately high rates of preventable skin cancers because of unaffordable sunscreen, inadequate dermatological services and the absence of health infrastructure in rural areas, factors that collectively reduce life expectancy and entrench avoidable morbidity (Luande et al. 1985; Standing Voice 2017; UTSS 2012).

Educational exclusion illustrates another domain where rights violations translate directly into lived disadvantage. Despite guarantees of inclusive education under the *CRC, CRPD* Article 24 and the *African Charter on the Rights and Welfare of the Child*, children with albinism frequently encounter schools that lack basic low-vision accommodations such as large-print materials, magnification devices, appropriate lighting or preferential seating. These institutional shortcomings, compounded by bullying and teacher misperceptions, often drive underachievement or early withdrawal despite children exhibiting typical cognitive ability (eds. Kromberg & Manga 2018; Standing Voice 2017). Violations of equality and non-discrimination norms, protected under the *ACHPR* and *CRPD* Article 5, are embedded in everyday social life. Persistent name-calling, stereotyping and social exclusion diminish participation in community life and limit access to employment and economic opportunities (Ero et al. 2021; OHCHR 2013).

For women with albinism, gendered forms of violence constitute an additional breach of rights under the *Maputo Protocol* and the *CRPD*. Sexual violence linked to the belief that intercourse with a woman with albinism can cure HIV or infertility reflects the convergence of disability-based stigma, gender discrimination and harmful cultural myths (OHCHR 2013; UTSS 2012, 2022). Finally, the right to access justice and obtain effective remedies, affirmed by the *ACHPR* and *CRPD* Article 13, is weakened by systemic failures, including delayed investigations, lenient sentences, inadequate victim support and weak institutional accountability (Ero 2019; OHCHR 2013). These deficiencies not only impede redress but also perpetuate cycles of violence by diminishing deterrence and eroding trust in state institutions.

Taken together, these patterns underscore how violations across different rights domains reinforce one another: violence restricts mobility and schooling; educational exclusion compounds poverty; health neglect reduces lifespan; and impunity sustains fear. Understood through CDS, these harms are not isolated incidents but reflect entrenched structural conditions requiring coordinated state action, preventive measures and deep cultural transformation.

### Advocacy and the struggle for recognition

For decades, the rights of PWAs received little sustained attention from international bodies, national governments or even the wider disability rights movement. References in early UN documents were sporadic, usually buried within discussions of racial discrimination, child rights or public health (OHCHR 2013). This neglect reflects what Fraser (2000) terms the politics of recognition: marginalised groups often remain invisible until crises force their acknowledgement, yet recognition alone rarely guarantees structural transformation. The trajectory of albinism advocacy has therefore been one of belated visibility: long periods of silence punctuated by moments of international outcry when killings or abuses became too visible to ignore.

A turning point came in the early 2000s, when reports of ritual killings in Tanzania, Malawi, Mozambique and Burundi drew international condemnation (Ero 2019). Persons with albinism were being hunted, mutilated and killed for their body parts, believed to bring wealth or power in witchcraft practices (Franklin et al. 2018; OHCHR 2013). Media coverage and NGO documentation generated widespread outrage, thrusting albinism into the international spotlight as a distinct human rights concern. In 2013, six UN special procedures issued a joint statement condemning these atrocities and urging African governments to act, while the African Commission on Human and Peoples’ Rights (2013) recognised the situation in Kenya and Tanzania as a pressing regional concern. These interventions were significant not only because they condemned violence but also because they repositioned albinism within existing human rights frameworks, making clear that PWAs are rights-holders under instruments such as the *ACHPR* and the *CRPD*.

International advocacy continued to expand. In 2015, the UN Human Rights Council created the mandate of the Independent Expert on the Enjoyment of Human Rights by Persons with Albinism, the first dedicated mechanism for monitoring the rights of PWAs. Led by Ikponwosa Ero, herself a woman with albinism, the mandate was ground-breaking in providing visibility, expert knowledge and international accountability (Ero 2019). It documented violations, issued thematic reports on health, education and security, and provided guidance to states on best practices (Ero et al. 2021). That same year, the UN proclaimed 13 June as International Albinism Awareness Day, offering a global focal point for mobilisation and education (OHCHR 2015). These developments signalled that albinism had finally been reframed as a matter of international concern and a human rights priority.

Yet the most transformative changes have come from grassroots mobilisation within Africa. Civil society organisations have often led the way. Groups such as Under the Same Sun and Standing Voice have raised awareness of ritual killings, promoted access to sunscreen and protective gear, and advocated for inclusive education (Standing Voice 2017; UTSS 2012). Their campaigns highlighted the urgent need to protect PWAs from violence and to dismantle structural barriers that keep them at society’s margins. Similarly, national associations of PWAs, such as the Tanzania Albinism Society, have provided platforms for self-advocacy, insisting that PWAs speak not only as victims but also as agents of change (eds. Kromberg & Manga 2018). In Malawi, the Association of Persons with Albinism lobbied for criminal accountability in cases of ritual killings, while in Kenya and Zimbabwe, advocacy groups have used media campaigns, public demonstrations and cultural events to challenge stigma (Ero 2019).

Globally, the number of advocacy organisations addressing albinism has increased markedly in the past two decades (Ero et al. 2021). The internet has been a crucial tool, enabling PWAs to connect across borders, share experiences and craft collective identities where isolation once prevailed. Digital activism has reshaped the geography of advocacy, allowing individuals in rural or marginalised settings to participate in global networks. The establishment of the Global Albinism Alliance (GAA) in 2020 consolidated these trends (Ero et al. 2021). For the first time, civil society organisations representing PWAs from six continents created a formal coalition to coordinate advocacy, share strategies and amplify visibility internationally. The GAA represents more than symbolic unity; it institutionalises global solidarity, reframes albinism from a localised issue into a transnational disability rights movement, and demonstrates how grassroots actors can reshape international agendas (Ero 2019; Ero et al. 2021).

Alongside international advocacy, national and community-based organisations have provided critical leadership. The Tanzania Albinism Society, established in the 1970s, remains one of the most influential grassroots organisations, offering psychosocial support, lobbying for protective policies and challenging discriminatory practices (eds. Kromberg & Manga 2018). In Uganda, the Albinism Umbrella (2023) has advanced public awareness campaigns and contributed to the election of parliamentarians with albinism, symbolising a breakthrough in political representation. In Malawi, the Association of Persons with Albinism has combined legal challenges with survivor support (Nation Online 2023). These examples illustrate how African PWAs themselves are shaping the discourse, transforming narratives of victimhood into claims of agency and leadership (OHCHR 2013).

Women with albinism face compounded vulnerabilities, particularly in contexts where myths sexualise or instrumentalise their bodies. Reports from Malawi and Tanzania document cases of sexual assault driven by the belief that intercourse with a woman with albinism can cure HIV (OHCHR 2013; UTSS 2012, 2022). At the same time, advocacy by women’s groups has challenged these intersecting oppressions, bringing gender-specific issues into the broader disability rights movement. Campaigns such as Climb for Albinism in Kenya, which placed women with albinism at the forefront of advocacy, symbolise both the persistence of gendered stigma and the potential for agency and empowerment (Bhalla 2018).

These developments reveal recurring strategies and best practices. One impactful strategy has been coalition-building and global coordination. From local associations to transnational alliances, collective action has amplified visibility, facilitated exchange of strategies and enabled coordinated responses to cross-border issues such as trafficking in body parts. Yet sustaining such coalitions requires secure funding and careful balancing of global priorities with local realities (Ero 2019). Without strong anchoring in local contexts, global advocacy risks reproducing the top-down dynamics it seeks to challenge.

Policy engagement and state collaboration have also proved essential. In Nigeria, the Albino Foundation partnered with the federal government to provide free skin cancer treatment for PWAs and to shape inclusive education policy (Punch Healthwise 2025). In Tanzania, Standing Voice developed a sunscreen distribution initiative with local authorities, providing sustainable access to sun protection (Standing Voice 2017). These cases show that advocacy can produce institutionalised protections when NGOs and governments work together. But they also highlight an enduring tension: many such programmes remain heavily donor-dependent. If sunscreen distribution, cancer treatment or educational accommodations are provided primarily by NGOs, states risk abdicating their primary responsibility to guarantee fundamental rights. Advocacy must therefore navigate a delicate balance: partnering with governments to deliver immediate benefits while also holding those same governments accountable for systemic reforms.

Another critical strand of advocacy is public awareness and cultural transformation. Because stigma is rooted in deeply held myths and beliefs, legal reforms alone are insufficient. Advocacy groups have used creative strategies to shift cultural narratives. Annual International Albinism Awareness Day provides a global platform for education (OHCHR 2015). Beauty pageants such as Mr. and Miss Albinism in Kenya and Zimbabwe challenge stereotypes by celebrating visibility and self-esteem (Ero 2019). Campaigns like Climb for Albinism place women with albinism at the centre of highly visible events, challenging gendered stereotypes and cultural myths (Bhalla 2018). Artistic expression, including drama, poetry and music, has also been harnessed to counter stigma and generate empathy (Ero et al. 2021). These initiatives demonstrate the symbolic power of advocacy, but they also carry risks: without linkage to structural reforms in education, healthcare or justice, symbolic campaigns may be dismissed as performative.

Education and capacity-building are equally vital for long-term empowerment. In Burundi and Tanzania, advocacy groups have trained teachers to accommodate children with albinism, addressing both pedagogical needs and cultural stigma in classrooms (Standing Voice 2017). Scholarships and mentorship programmes have expanded access to higher education, creating pathways for PWAs to enter professions and leadership roles. These interventions directly challenge cycles of poverty and exclusion. Yet they are unevenly distributed, often concentrated in urban centres while rural communities remain neglected (eds. Kromberg & Manga 2018). This gap underscores the difficulty of scaling up pilot projects into systemic reforms.

Perhaps the most transformative trend has been the rise of self-representation and political participation. Persons with albinism increasingly lead advocacy organisations, shape agendas and hold decision-makers accountable. In Uganda, activism by the Albinism Umbrella contributed to the election of the first parliamentarian with albinism, a breakthrough in both visibility and substantive representation (Ero 2019). Across Africa, women with albinism have mobilised around gender-specific vulnerabilities, including sexual assault driven by myths that intercourse with them cures HIV (Ero et al. 2021). These examples show advocacy is not only about survival but also about transforming identity, agency and political presence.

Despite these achievements, advocacy faces enduring challenges and limitations. Visibility does not always translate into structural change. Awareness campaigns, symbolic events and international resolutions raise the profile of albinism, but do not automatically dismantle systemic barriers. Advocacy also remains heavily dependent on donor funding, raising concerns about sustainability once external attention declines (Ero 2019). This donor dependency intersects with Fraser’s (2000) observation that recognition without redistribution can entrench rather than resolve inequality. Persons with albinism are increasingly acknowledged in discourse, yet resources and reforms lag behind. Moreover, the prominence of NGOs can obscure state failures, effectively shifting responsibility for rights protection from governments to civil society.

From a scholarly perspective, advocacy for PWAs illustrates the dynamics of social movements that emerge from lived experiences of exclusion. It exemplifies how marginalised groups transform stigma into collective identity and rights-claiming, and how advocacy scales up from local survival strategies to global coalitions. It also demonstrates the importance of intersectionality. Advocacy that ignores the compounded vulnerabilities of women, rural dwellers or children risks reproducing hierarchies within the movement itself (Crenshaw 1989). Finally, it underscores the continuing challenge of epistemic injustice. Even within advocacy, PWA’s voices must be central. Where external actors dominate narratives, PWAs risk being spoken for rather than empowered to speak for themselves.

Advocacy has therefore served a dual function. On one level, it is a survival mechanism, providing immediate protection, health interventions and solidarity in contexts of violence. On another, it is a long-term strategy for inclusion and justice, reframing albinism within disability and human rights discourse. Its achievements are significant – securing international recognition, building global coalitions and reshaping cultural narratives. Its challenges are equally profound – donor dependency, limited state accountability and the persistence of deep-rooted myths. For disability studies, advocacy around albinism offers a valuable case study of both the possibilities and limitations of grassroots mobilisation. It shows how collective action can move from survival to systemic change, but also how fragile such progress remains without sustained state commitment and structural transformation.

### Limitations

The reliance on secondary sources imposes several limitations. Firstly, while human rights reports provide valuable data, they tend to focus on extreme cases of violence, which may obscure the everyday forms of discrimination that PWAs face. Secondly, historical and biomedical texts, while foundational, reflect the biases of their time and must be read critically. Thirdly, the absence of first-person narratives from PWAs highlights a broader epistemic problem: their voices remain underrepresented in both scholarship and policy.

These limitations, however, do not diminish the value of this analysis. Instead, they underscore the need for future research that is participatory, comparative and empirically grounded.

## Conclusion

The experiences of PWAs reveal the enduring intersection of biology, culture, and rights. From early accounts in antiquity that cast PWAs as anomalies, through the medicalisation and genetic cataloguing of the 19th and 20th centuries, to contemporary rights frameworks, PWAs have persistently been positioned as subjects of myth, pathology or spectacle rather than recognised as rightsholders. Scientific advances in genetics and ophthalmology have clarified the biological basis of albinism, yet they have not addressed the cultural myths and structural conditions that perpetuate exclusion. In this sense, the history of albinism illustrates how biomedical knowledge has often reinforced epistemic injustice, producing knowledge about PWAs without their participation.

This article has argued that albinism must be framed as both a disability and a human rights issue. The medical model clarifies biological risks such as visual impairment and heightened vulnerability to skin cancer, but fails to capture the social dynamics of exclusion. The social model shifts focus to stigma, discrimination and disabling environments, yet struggles to fully address the material consequences of impairment in African contexts of high ultraviolet exposure. The human rights model, embodied in the CRPD, provides a more holistic framework by recognising the interaction of biology and society while obligating states to dismantle barriers and ensure equal participation. Intersectional analysis further demonstrates how race, gender and poverty exacerbate vulnerability, particularly for women with albinism who face both gendered and disability specific violence.

Albinism’s human rights dimensions demonstrate the indivisibility of rights across legal and social domains. Violations such as ritual killings and physical attacks infringe on the right to life and dignity, while barriers to health care violate the right to health as guaranteed by the ICESCR. Educational exclusion further contravenes the CRC, disproportionately affecting children with albinism. The persistence of gendered violence, particularly toward women with albinism, highlights the need for gender-sensitive legal responses under frameworks like the Maputo Protocol. Despite advancements, such as the African Disability Protocol, implementation gaps persist, emphasising the disconnect between normative commitments and lived realities.

Advocacy has been instrumental in reshaping the landscape of albinism, with grassroots organisations providing critical support and international recognition – such as the UN Independent Expert on Albinism – drawing attention to the global dimensions of the issue. While these efforts have enhanced visibility, they remain constrained by challenges like donor dependency and inconsistent state responses. Advocacy’s dual role, both as a survival mechanism and a strategy for systemic change, illustrates the need for comprehensive, structural interventions.

This study contributes to disability scholarship by positioning albinism as a key case to interrogate the intersections of disability, race and human rights. It foregrounds epistemic injustice, demonstrating how the exclusion of PWAs from data, policy and scholarship perpetuates marginalisation. By emphasising intersectional, rights-based frameworks, this work underscores the importance of capturing the layered realities of disability in African contexts and challenging epistemic blind spots in both scholarship and policy.

For African disability scholarship, albinism represents a critical test case, revealing the marginalisation of PWAs within the broader field of disability studies. The experiences of PWAs, at the intersection of race, skin, gender and poverty, demand inclusive frameworks that address cultural specificities of exclusion and stigma. Recognising albinism as both a disability and a human rights issue affirms the need for African-led approaches, rather than imported paradigms, to disability studies.

This article also highlights the resilience and agency of PWAs in the face of systemic violence and exclusion. African disability studies must address the cultural legacies of stigma and the epistemic injustices that silence marginalised groups. By doing so, it can create more inclusive, contextually grounded and justice-oriented frameworks. For policy and practice, three imperatives emerge: strengthening legal protections against violence, expanding access to health and education (including sunscreen and inclusive schooling), and institutionalising the representation of PWAs in policymaking. These measures, mandated by international and regional law, are not optional. Future research should address gaps in data, particularly through large-scale, disaggregated surveys and qualitative studies to capture the lived experiences of PWAs. Comparative studies across regions would also deepen understanding of how albinism is constructed and addressed across different cultural and institutional settings, ensuring that PWA’s voices are central in knowledge production.

### Analytical contribution of critical disability studies

By mobilising CDS, this article reframes albinism from an object of clinical fascination or cultural myth to a site of structural disablement and rights contestation. Critical disability studies clarifies: (1) how medicalisation and data invisibility constitute epistemic injustice; (2) how material deprivation (e.g. sunscreen) and institutional neglect transform impairment into avoidable disability; and (3) how advocacy functions as both counter knowledge and redistributive politics, moving recognition beyond symbolism toward enforceable obligations. This theoretical lens supports normative claims (state duties under CRPD and African instruments) and provides a diagnostic for policy design (e.g. data systems, inclusive education, accessible health) (Amnesty International 2025).

Ultimately, the story of albinism is one of both vulnerability and resilience, exposing the costs of exclusion while offering a blueprint for advancing justice. Recognising PWAs as rightsholders is not only a moral imperative but a test of the global human rights project’s credibility.
